# Dietary Exposure to Antibiotic Residues Facilitates Metabolic Disorder by Altering the Gut Microbiota and Bile Acid Composition

**DOI:** 10.1128/msystems.00172-22

**Published:** 2022-06-07

**Authors:** Rou-An Chen, Wei-Kai Wu, Suraphan Panyod, Po-Yu Liu, Hsiao-Li Chuang, Yi-Hsun Chen, Qiang Lyu, Hsiu-Ching Hsu, Tzu-Lung Lin, Ting-Chin David Shen, Yu-Tang Yang, Hsin-Bai Zou, Huai-Syuan Huang, Yu-En Lin, Chieh-Chang Chen, Chi-Tang Ho, Hsin-Chih Lai, Ming-Shiang Wu, Cheng-Chih Hsu, Lee-Yan Sheen

**Affiliations:** a Institute of Food Science and Technology, National Taiwan University grid.19188.39grid.412094.a , Taipei, Taiwan; b Department of Medical Research, National Taiwan University grid.19188.39grid.412094.a Hospital, Taipei, Taiwan; c Department of Internal Medicine, College of Medicine, National Taiwan University grid.19188.39grid.412094.a , Taipei, Taiwan; d National Laboratory Animal Center, National Applied Research Laboratories, Taipei, Taiwan; e Department of Chemistry, National Taiwan University grid.19188.39grid.412094.a , Taipei, Taiwan; f The Metabolomics Core Laboratory, Center of Genomic Medicine, National Taiwan University grid.19188.39grid.412094.a , Taipei, Taiwan; g Department of Medical Biotechnology and Laboratory Science, College of Medicine, Chang Gung University, Taoyuan, Taiwan; h Division of Gastroenterology, Perelman School of Medicine, University of Pennsylvania, Philadelphia, Pennsylvania, USA; i Department of Internal Medicine, National Taiwan University grid.19188.39grid.412094.a Hospital, Taipei, Taiwan; j Department of Food Science, Rutgers University, New Brunswick, New Jersey, USA; k Center for Food and Biomolecules, National Taiwan University grid.19188.39grid.412094.a , Taipei, Taiwan; l National Center for Food Safety Education and Research, National Taiwan University grid.19188.39grid.412094.a , Taipei, Taiwan; Duke University

**Keywords:** bile acid metabolism, dietary exposure, early life, food safety, gut microbiota, low-dose antibiotic, metabolic disorder, obesity

## Abstract

Antibiotics used as growth promoters in livestock and animal husbandry can be detected in animal-derived food. Epidemiological studies have indicated that exposure to these antibiotic residues in food may be associated with childhood obesity. Herein, the effect of exposure to a residual dose of tylosin—an antibiotic growth promoter—on host metabolism and gut microbiota was explored *in vivo*. Theoretical maximal daily intake (TMDI) doses of tylosin were found to facilitate high-fat-diet-induced obesity, induce insulin resistance, and perturb gut microbiota composition in mice. The obesity-related phenotypes were transferrable to germfree recipient mice, indicating that the effects of a TMDI dose of tylosin on obesity and insulin resistance occurred mainly via alteration of the gut microbiota. Tylosin TMDI exposure restricted to early life, the critical period of gut microbiota development, altered the abundance of specific bacteria related to host metabolic homeostasis later in life. Moreover, early-life exposure to tylosin TMDI doses was sufficient to modify the ratio of primary to secondary bile acids, thereby inducing lasting metabolic consequences via the downstream FGF15 signaling pathway. Altogether, these findings demonstrate that exposure to very low doses of antibiotic residues, whether continuously or in early life, could exert long-lasting effects on host metabolism by altering the gut microbiota and its metabolites.

**IMPORTANCE** This study demonstrates that even with limited exposure in early life, a residual dose of tylosin might cause long-lasting metabolic disturbances by altering the gut microbiota and its metabolites. Our findings reveal that the gut microbiota is susceptible to previously ignored environmental factors.

## INTRODUCTION

Antibiotics administered at subtherapeutic doses have been used as growth promoters since the 1940s (1–3). Livestock fed with antibiotic growth promoter (AGP) exhibited improved feed utilization, decreased infection, and increased weight gain, bringing substantial economic benefits in meat-producing countries (1, 2, 4). According to the U.S. Food and Drug Administration, approximately two-thirds of all antimicrobial agents used in the United States are for livestock and animal husbandry, driven by the demand to improve the production of animal-derived foods (5). To avoid potential health hazards to consumers, the Joint FAO/WHO Expert Committee on Food Additives evaluated and provided maximum residue limits (MRLs) for veterinary antibiotic residues permitted in food (6). The MRLs were determined based on acceptable daily intake (ADI) that should be harmless to humans according to results from extensive toxicity studies. However, these studies did not evaluate animal models of chronic metabolic diseases. They were designed without considering the effects of antibiotic residues on gut microbiota and microbial metabolites, thereby failing to establish levels that can affect the gut microbiota, and thus the health of the host, and cause disease.

Although AGP has been prohibited in some countries, studies reported that antibiotics remained in meat, eggs, milk, and seafood products (7–9), sometimes even at levels exceeding the MRLs (10). Moreover, cooking processes, such as frying and roasting, can increase the concentrations of certain antibiotics (8), raising the probability of exposure to antibiotic residues through food. Therefore, veterinary antibiotics can be detected in human urine due to the consumption of pork, chicken, and dairy products (9). Furthermore, higher levels of veterinary antibiotics detected in the urine were reported to positively correlate with obesity in children, revealing that exposure to antibiotic residues in food may contribute to obesity (11).

The commensal bacteria play a crucial role in human health and disease mainly by producing various metabolites, such as short-chain fatty acids (SCFAs) and secondary bile acids (12). Indeed, dysbiosis of the gut microbiota and microbial metabolites have been associated with metabolic diseases (13, 14). Antibiotics significantly disturb the composition of the gut microbiota and alter SCFAs and bile acids, as well as their signaling pathways, thereby leading to metabolic consequences (15–17). Hence, considering their importance in treating infections, antibiotics can be viewed as a double-edged sword for human health, given their untoward effects on the gut microbiota and host metabolic homeostasis (18).

The gut microbial community is dynamic and susceptible to environmental shifts in early life (19). Growing evidence indicates that antibiotic administration in early life is associated with an increased risk of being overweight and obese (20–23). More specifically, antibiotic exposure during early life, even at subtherapeutic levels, disturbs the colonization and maturation of the intestinal microbiota, leading to lasting effects on the metabolism of the host (19, 24). Moreover, antibiotics have no growth-promoting effects in germ-free chickens (25), indicating that the effects are driven by the alternation of the gut microbiota. Along with the above-mentioned findings in an epidemiological study (11), low-dose veterinary antibiotic residues in food may promote obesity via gut microbiota perturbation. However, the impact of residual doses of antibiotics on the gut microbiota and human health has not been elucidated (3, 26).

In this study, we investigated whether chronic exposure to acceptable residual amounts of antibiotics, whose doses are a twenty-fold lower than the subtherapeutic doses used in previous studies, could cause obesity complications. To study the effect of tylosin-altered microbiotas on obesity-related phenotypes, fecal samples were transplanted from mice fed a theoretical maximal daily intake (TMDI) dose of tylosin into germfree mice. In addition, whether early life exposure to TMDI doses of tylosin could induce obesity-related complications and cause alterations in gut microbiotas and microbial metabolites was also investigated. Finally, we identified a plausible mechanism by which tylosin TMDI doses induced metabolic consequences via altering bile acid composition and the ileal fibroblast growth factor 15 (FGF15)/hepatic fibroblast growth factor receptor 4 (FGFR4) pathway.

## RESULTS

### A residual dose of tylosin facilitate obesity preferentially in high-fat-diet-fed mice.

**FIG 1:**
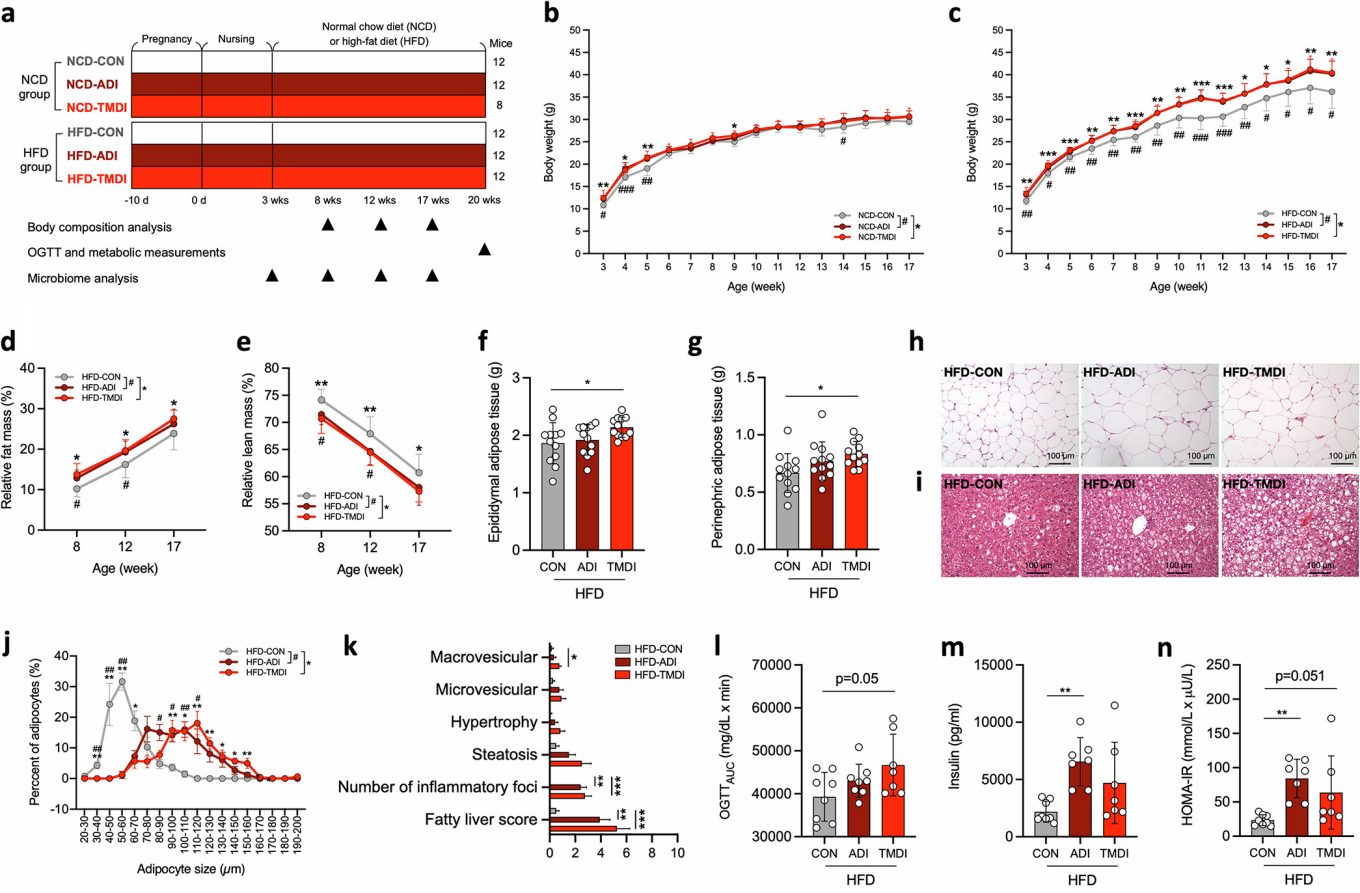
Residual dose of tylosin facilitates HFD-induced obesity and metabolic disorders. (a) Experimental design of antibiotic residue exposure model. (b) Body weight changes in NCD (*n* = 12, 12, and 8 mice per group) and (c) HFD-fed mice (*n* = 12 mice per group). (d) Relative fat mass and (e) relative lean mass changes in HFD mice (*n* = 12 mice per group). (f) Weight of epididymal adipose tissue and (g) perinephric adipose tissue (*n* = 12 mice per group). (h) Representative histological features of H&E stained epididymal adipose tissue and (i) and liver. (j) Adipocyte diameter of epididymal adipose tissue (*n* = 6 mice per group). (k) Fatty liver score, including steatosis (macrovesicular, microvesicular, and hypertrophy) and inflammation (number of inflammatory foci) (*n* = 12, 12, and 8 mice per group). (l) Area under the curve (AUC) derived from the OGTT (*n* = 8, 8, and 7 mice per group). (m) Plasma insulin level after overnight fasting (*n* = 8, 7, and 7 mice per group). (n) HOMA-IR index represented as an indicator of insulin resistance (*n* = 8, 7, and 7 mice per group). Data are means and SD. For panels b to e and j, statistical analyses were performed by one-way ANOVA with Tukey’s range test within diet groups (NCD or HFD) as follows: CON versus ADI (*#*, *P* < 0.05; #*#*, *P* < 0.01); CON versus TMDI (*, *P* < 0.05; **, *P* < 0.01; ***, *P* < 0.001). For panels f, g, and k to n, one-way ANOVA with Tukey’s range test was performed (*, *P* < 0.05; **, *P* < 0.01; ***, *P* < 0.001). Abbreviations: ADI, acceptable daily intake; AUC, area under the curve; CON, control; HFD, high-fat diet; HOMA-IR, homeostatic model assessment of insulin resistance; NCD, normal chow diet; OGTT, oral glucose tolerance test; TMDI, theoretical maximum daily intake.

To investigate whether chronic exposure to an acceptable or residual dose of antibiotic could cause obesity, and to examine the potential synergistic effects of antibiotics and diet on host metabolism, a murine model that included two doses of the antibiotic tylosin (ADI, 0.37 mg/kg; TMDI, 0.047 mg/kg) and two different diets (normal chow diet [NCD] and high-fat diet [HFD]) were designed (Fig. 1a). The ADI dose is the maximum daily dose posing no adverse effect (6), while the TMDI dose is an estimate of the maximum dietary intake level obtained for MRLs and the sum of average daily per capita consumption for each food product (6), which was used for simulating the ingestion of antibiotic residues through food consumption in the present study.

Compared with NCD-CON (nonexposed control) mice, NCD-ADI and NCD-TMDI mice showed significantly greater weight gain from weaning to 5 weeks of age (Fig. 1b), with no significant changes detected among the NCD groups in relative fat or lean mass (Fig. S1a and b). In contrast, HFD-ADI and HFD-TMDI mice exhibited significantly increased weight gain compared with the HFD-CON group throughout the experiment from weaning to 17 weeks of age (Fig. 1c). HFD-ADI and HFD-TMDI mice had increased relative fat mass at weeks 8, 12, and 17, compared with HFD-CON mice (Fig. 1d), whereas HFD-TMDI mice had decreased relative lean mass (Fig. 1e). These results demonstrate that tylosin-induced adiposity is evident early in life with both NCD and HFD. Still, the continuous effect of tylosin-induced adiposity was preferentially observed in HFD-fed mice.

10.1128/msystems.00172-22.1 FIG S1 Effects of tylosin on body composition and adipose tissue. (a) Relative fat mass and (b) relative lean mass change of NCD mice (*n* = 12 mice per group). (c) Size of epididymal and perirenal fat. (d) Size of inguinal fat. Data are means and SD. Statistical analyses were performed by one-way ANOVA with Tukey’s range test. Abbreviations: ADI, acceptable daily intake; CON, control; HFD, high-fat diet; NCD, normal chow diet; TMDI, theoretical maximum daily intake. Download FIG S1, TIF file, 2.4 MB. Copyright © 2022 Chen et al. 2022 Chen et al. https://creativecommons.org/licenses/by/4.0/ This content is distributed under the terms of the Creative Commons Attribution 4.0 International license.

### A residual dose of tylosin exacerbate HFD-induced hepatic steatosis, adiposity, and insulin resistance.

Antibiotics have been shown to induce obesity, nonalcoholic fatty liver disease (NAFLD), and insulin resistance (27). We next sought to investigate whether exposure to residual tylosin dose is causally associated with metabolic complications, including hepatic abnormalities and glucose homeostasis. Given that tylosin increased the fat mass in HFD-fed mice but not in NCD-fed mice, additional investigations in the HFD-fed mice were conducted. Likewise, HFD-TMDI mice had increased visceral fat, including epididymal and perinephric adipose tissues (*P* = 0.0486 and *P* = 0.0302, respectively) (Fig. 1f and g and Fig. S1c and d). HFD-ADI and HFD-TMDI mice also exhibited adipocyte hypertrophy (Fig. 1h) and elevated adipocyte size (Fig. 1j) compared with HFD-CON mice.

Histological examination of the liver revealed that tylosin-treated mice exhibited increased lipid droplet formation (Fig. 1i), more inflammatory foci, and higher fatty liver scores (Fig. 1k), suggesting that the presence of residual tylosin caused more severe NAFLD. Both HFD-ADI and HFD-TMDI mice exhibited a trend toward higher plasma glucose during the oral glucose tolerance test (OGTT) (Fig. S2a) and fasting glucose test (Fig. S2b), with an increased area under the curve for the OGTT (OGTT_AUC_) in HFD-TMDI mice (Fig. 1l). The fasting insulin level and homeostasis model assessment of insulin resistance (HOMA-IR) index were also elevated in HFD-ADI mice compared with HFD-CON mice (*P* = 0.0058 and *P* = 0.0075, respectively) (Fig. 1m and n). These results reveal that even tylosin residues can induce adverse effects on metabolism when an HFD is consumed. Remarkably, although the tylosin ADI dose is defined as the maximum antibiotic dose that can be ingested daily without health risk (6), it facilitated obesity-related metabolic disorders in our mouse model.

10.1128/msystems.00172-22.2 FIG S2 Effects of tylosin on metabolic parameters. (a) Plasma glucose profile measured during the OGTT (*n* = 8, 8, and 7 mice per group). (b) Plasma glucose after overnight fasting (*n* = 8, 8, and 7 mice per group). (c) Plasma triglycerides and (d) total cholesterol level after overnight fasting (*n* = 9 mice per group). (e) Average food intake (*n* = 4 mice per group). (f) Basal energy expenditure (*n* = 4 mice per group). (g) Plasma lipopolysaccharides level (*n* = 9, 8, and 8 mice per group). Data are means and SD. Statistical analyses were performed by one-way ANOVA with Tukey’s range test (*, *P < *0.05). Abbreviations: ADI, acceptable daily intake; CON, control; HFD, high-fat diet; TMDI, theoretical maximum daily intake. Download FIG S2, TIF file, 0.4 MB. Copyright © 2022 Chen et al. 2022 Chen et al. https://creativecommons.org/licenses/by/4.0/ This content is distributed under the terms of the Creative Commons Attribution 4.0 International license.

### Exposure to residual doses of tylosin alter the gut microbiota composition.

As subtherapeutic doses of antibiotic treatments have been shown to disrupt the development and maturation of gut microbiota with metabolic consequences (19, 28), we next asked whether ADI and TMDI doses of tylosin alter gut microbiota composition. To this end, fecal microbiota analysis was performed to elucidate changes in the gut microbiota in mice at 3 (weaning), 8, and 17 weeks of age by using 16S amplicon sequence variants (ASVs).

**FIG 2:**
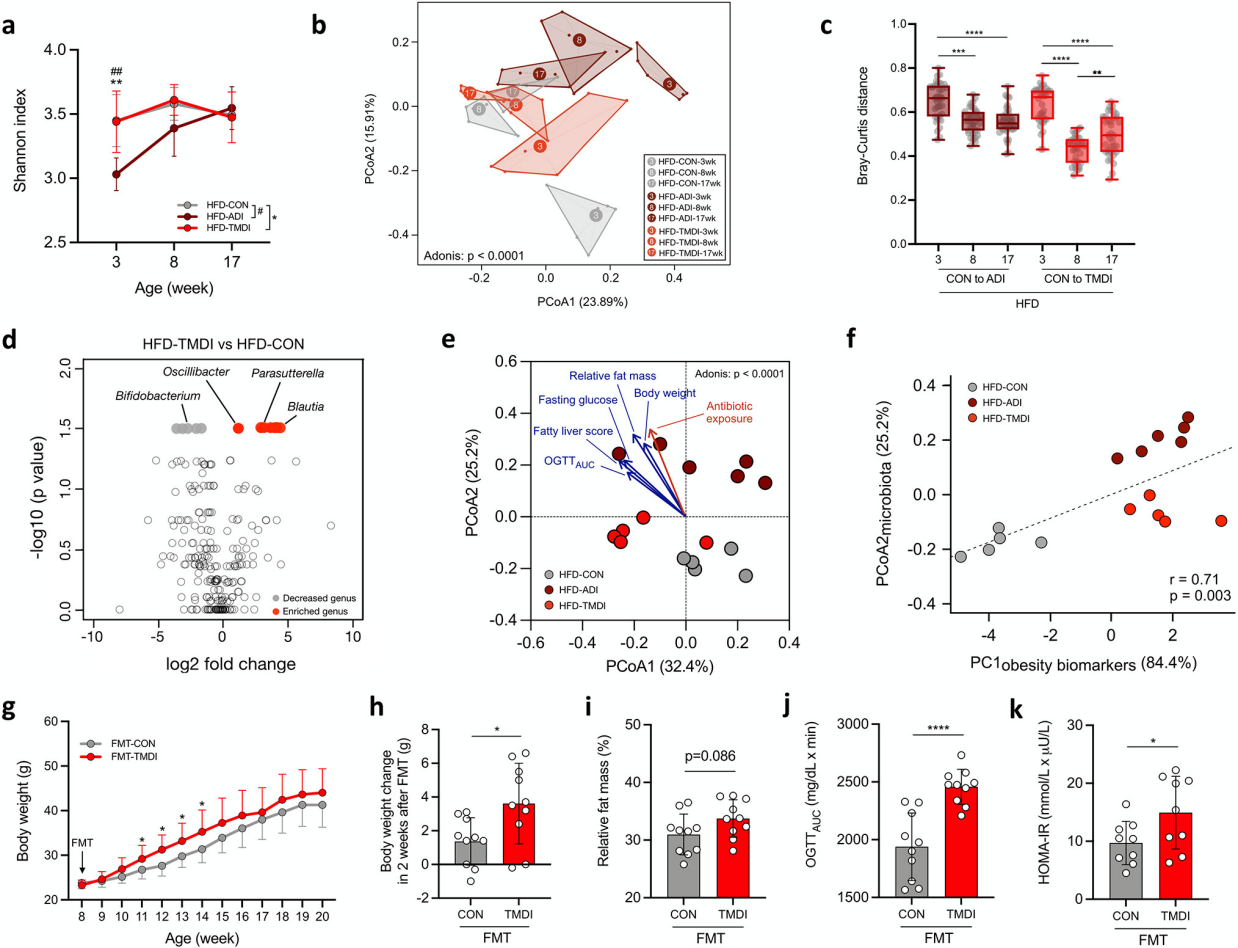
Residual dose of tylosin remodels the gut microbiota composition, and their microbiota shift verifies its pathogenesis on generating the obesogenic and metabolic phenotype in germfree mice. (a) Change in Shannon diversity index in 3-, 8-, and 17-week-old HFD-fed mice (*n* = 6 mice per group). (b) Gut microbiota composition as represented by principal-coordinate analysis (PCoA) of Bray-Curtis distances for 3-, 8-, and 17-week-old HFD-fed mice (*n* = 6 mice per group). The PCoA analysis of data from both HFD and NCD mice is shown in Fig. S3a. (c) Bray-Curtis dissimilarity index comparing distances of tylosin-treated groups to CON at different time points (*n* = 6 mice per group). (d) Volcano plot showing bacterial taxa whose abundance was increased or decreased (based on a log_2_ fold change of >1) and significant difference (*P* < 0.05) estimated using the Wilcoxon signed-rank test (*n* = 6 mice per group). (e) Bray-Curtis distance-based PCoA of 17-week-old HFD-fed mice’s gut microbiota and the fitted antibiotic exposure/obesity-related variables, which significantly correlated with the shifted microbiome (*P* < 0.05) using the envfit package in R (*n* = 5, 5, and 6 mice per group). (f) Spearman’s correlation of PCoA2_microbiota_ and PC1_obesity biomarkers_ (*n* = 5, 5, and 6 mice per group). (g) Body weight change in germ-free mice transplanted with feces of HFD-CON and HFD-TMDI mice (*n* = 10 mice per group). (h) Body weight change 2 weeks posttransplantation (*n* = 10 mice per group). (i) Relative fat mass at 20 weeks of age (*n* = 10 mice per group). (j) AUC derived from the OGTT (*n* = 9 mice per group). (k) HOMA-IR index (*n* = 9 mice per group). Data are means and SD. For panel a, statistical analyses were performed with the Wilcoxon signed-rank test as follows: HFD-CON versus HFD-ADI (#*#*, *P* < 0.01) and HFD-CON versus HFD-TMDI (**, *P* < 0.01). For panel b and e, Adonis was performed to test the difference among groups. For panel c, one-way ANOVA with Tukey’s range test was performed (**, *P* < 0.01; ***, *P* < 0.001; ****, *P* < 0.0001). For panel e, envfit was performed to fit obesity-related variables and tylosin doses onto PCoA ordination of gut microbiota composition. For panels g to k, unpaired two-tailed *t* test was performed (*, *P* < 0.05; ****, *P* < 0.0001). Abbreviations: AUC, area under the curve; CON, control; FMT, fecal microbiota transplantation; HOMA-IR, homeostatic model assessment of insulin resistance; OGTT, oral glucose tolerance test; TMDI, theoretical maximum daily intake.

Compared with HFD-TMDI and HFD-CON mice, HFD-ADI mice had significantly reduced Shannon indices at week 3, which markedly increased during weeks 3 to 17 (Fig. 2a). Principal coordinate analysis (PCoA) based on Bray-Curtis dissimilarity further revealed that the microbiomes of NCD and HFD mice clustered separately (Adonis; *P* < 0.0001) (Fig. S3a), indicating that diet may represent the most critical factor influencing the gut microbiota composition, while residual tylosin dose exhibited a secondary effect in shifting the gut microbiota in a dose-dependent manner according to the PCoA2 axis (Fig. S3a).

10.1128/msystems.00172-22.3 FIG S3 Effects of diet, tylosin, and age on gut microbiota composition. Bacterial communities represented by principal-coordinate analysis (PCoA) of Bray-Curtis distances of (a) effects of diet and tylosin on gut microbiota composition (*n* = 6 mice per group) and (b) effects of tylosin in NCD-fed mice at 3, 8, and 17 weeks old (*n* = 6 mice per group). Adonis was performed to test the difference among groups. Abbreviations: ADI, acceptable daily intake; CON, control; HFD, high-fat diet; NCD, normal chow diet; TMDI, theoretical maximum daily intake. Download FIG S3, TIF file, 0.6 MB. Copyright © 2022 Chen et al. 2022 Chen et al. https://creativecommons.org/licenses/by/4.0/ This content is distributed under the terms of the Creative Commons Attribution 4.0 International license.

Further, we separately analyzed the subsets of NCD-fed mice (Fig. S3b) and HFD-fed mice (Fig. 2b) to observe the effect of tylosin on age-related microbiota development (Adonis; *P* < 0.0001). In both groups, the ADI dose of tylosin substantially influenced the gut microbiota composition, while the TMDI dose of tylosin showed a lesser impact. Notably, the gut microbiota of tylosin-treated mice was expectedly changed at the early life stage (week 3) in both NCD and HFD groups, gradually shifting toward the control group at weeks 8 and 17, respectively. Bray-Curtis distances from HFD-CON mice to HFD-ADI or HFD-TMDI mice were also shortened as the mice aged (Fig. 2c). These findings suggest that gut microbiota has increased susceptibility to the perturbation caused by residual tylosin in early life and that susceptibility may decrease at later stages, possibly due to the establishment and maturation of gut microbiota.

To further investigate bacteria whose abundance is differentially modified by the tylosin TMDI dose, we identified bacterial taxa whose abundance is significantly altered using the Wilcoxon signed-rank test (defined as a log_2_ fold change of >1, with *P* values of <0.05) (Fig. 2d; Fig. S4) and ANOVA (analysis of variance)-like differential expression analysis version 2 (ALDEx2) (defined as a Benjamini-Hochberg adjusted *P* value of <0.1) (Fig. S5) (29) at the level of ASVs. The abundance of *Oscillibacter*, *Blautia*, and *Parasutterella*, which are associated with obesity and inflammatory bowel disease (30–32), was significantly enriched in HFD-TMDI mice, whereas that of *Turicibacter*, *Faecalibaculum*, and *Bifidobacterium*, which are negatively associated with obesity (33–35), was significantly reduced. Collectively, the TMDI dose of tylosin may decrease the abundance of beneficial bacteria while increasing that of pathogenic bacteria.

10.1128/msystems.00172-22.4 FIG S4 Comparison fecal microbiota of HFD-CON and HFD-TMDI at ASV level. Heatmap showing bacteria with significant differences (*P* < 0.05) based on log_2_ fold change >1 and significant difference (*P* < 0.05) using the Wilcoxon signed-rank test (*n* = 6 mice per group). Abbreviations: CON, control; HFD, high-fat diet; TMDI, theoretical maximum daily intake. Download FIG S4, TIF file, 0.2 MB. Copyright © 2022 Chen et al. 2022 Chen et al. https://creativecommons.org/licenses/by/4.0/ This content is distributed under the terms of the Creative Commons Attribution 4.0 International license.

10.1128/msystems.00172-22.5 FIG S5 Comparison fecal microbiota of HFD-CON and HFD-TMDI using the ANOVA-like differential expression analysis version 2 (ALDEx2) at ASV level. (a) Effect size plot showing the median log_2_ difference against dispersion and (b) MA plot showing the median log_2_ difference against relative abundance of bacteria of HFD-CON versus HFD-TMDI mice at ASV level. (c) Eleven bacteria significantly enriched or decreased in HFD-TMDI mice and their corresponding taxa names. The analysis was performed by using ALDEx2 framework with a Benjamini-Hochberg-adjusted *P* value of <0.1 (*n* = 6 mice per group). The red plots indicate significantly altered ASVs. Abbreviations: CON, control; HFD, high-fat diet; TMDI, theoretical maximum daily intake. Download FIG S5, TIF file, 0.7 MB. Copyright © 2022 Chen et al. 2022 Chen et al. https://creativecommons.org/licenses/by/4.0/ This content is distributed under the terms of the Creative Commons Attribution 4.0 International license.

We next assessed the association between obesity-related features of 17-week-old HFD-fed mice and their gut microbiota composition by the envfit function in the vegan R package; the result is displayed as a PCoA (Fig. 2e). The results showed that vectors indicating both obesity-related biomarkers (the blue arrows) and antibiotic exposure (the red arrow) were directed toward the mice treated with tylosin in the same direction, indicating that the obesity outcomes were associated with the tylosin-shifted gut microbiome. We further performed Spearman’s correlation analysis to identify a correlation between PCoA1 and PCoA2 of the gut microbiota and individual obesity-related parameters (Table S1). We found that the PCoA2 was significantly correlated with obesogenic phenotypes (fat gain, fat mass, and relative fat mass), fatty liver score, and insulin resistance parameters (fasting insulin and HOMA-IR).

10.1128/msystems.00172-22.8 TABLE S1 Spearman’s correlation between obesity-related biomarkers against gut microbiota composition represented by PCoA1 and PCoA2. a, PCoA1 and PCoA2 were calculated based on Bray-Curtis distances of the 17-week-old HFD-fed mice’s gut microbiota. The correlation was calculated by Spearman’s correlation with FDR-adjusted *P* value. b, Abbreviations: AUC, area under the curve; HOMA-IR, homeostasis model assessment of insulin resistance; OGTT, oral glucose tolerance test; PCoA, principal-coordinate analysis. Download Table S1, DOCX file, 0.01 MB. Copyright © 2022 Chen et al. 2022 Chen et al. https://creativecommons.org/licenses/by/4.0/ This content is distributed under the terms of the Creative Commons Attribution 4.0 International license.

To understand the overall obesity phenotype and its relationship with the tylosin-altered microbiome, we performed a dimension reduction of the obesity- and metabolic disorder-related biomarkers using principal-component analysis (PCA). The result showed that the obesity phenotypes in tylosin-treated mice significantly differed from those of control mice (*P* < 0.0001) (Fig. S6). We subsequently evaluated the correlation between PC1_obesity biomarkers_ and PCoA2_microbiota_ and determined a positive correlation (*r* = 0.71, *P* = 0.003).

10.1128/msystems.00172-22.6 FIG S6 Principal-component analysis of the obesity biomarkers of mice. PCA of the obesity biomarkers of 17-week-old HFD-fed mice (*n* = 5, 6, and 5 mice per group). The PCA plot was calculated from obesity biomarkers, including body weight at 17 weeks of age, fat mass at 17 weeks of age, relative fat mass at 17 weeks of age, weight gain between 3 and 17 weeks of age, fat mass gain between 8 to 17 weeks of age, fasting glucose, OGTT_AUC_, fasting insulin, HOMA-IR, weight of eWAT, and fatty liver score. Adonis was performed to test the difference among groups. Abbreviations: ADI, acceptable daily intake; AUC, area under the curve; CON, control; HFD, high-fat diet; HOMA-IR, homeostasis model assessment of insulin resistance; OGTT, oral glucose tolerance test; TMDI, theoretical maximum daily intake. Download FIG S6, TIF file, 0.1 MB. Copyright © 2022 Chen et al. 2022 Chen et al. https://creativecommons.org/licenses/by/4.0/ This content is distributed under the terms of the Creative Commons Attribution 4.0 International license.

### Fecal microbiota transplantation from HFD-TMDI mice induces adiposity and insulin resistance in germfree mice.

Given the above association between the metabolic disorder phenotypes and tylosin-altered gut microbiota, we conducted a fecal microbiota transplantation (FMT) study to investigate their causative role. Since a TMDI dose can adequately simulate human exposure to antibiotics in food, feces from HFD-TMDI mice were transplanted to germfree mice. Compared with germfree mice that received feces from HFD-CON mice (FMT-CON), the HFD-TMDI recipient mice (FMT-TMDI) showed higher body weight at 11, 12, 13, and 14 weeks of age (Fig. 2g), increased weight gain 2 weeks post-FMT (*P* = 0.0195) (Fig. 2h), and slightly elevated fat mass at week 20 (*P* = 0.0861) (Fig. 2i), implying that the microbiome from HFD-TMDI mice increased the adiposity of the recipient mice. Additionally, FMT-TMDI mice exhibited increased plasma glucose levels in OGTT_AUC_ and a higher HOMA-IR index (*P* < 0.0001 and *P* = 0.0472, respectively) (Fig. 2j and k). Thus, the gut microbiota altered by TMDI-dose tylosin induced obesity and insulin resistance in germfree recipients, indicating that the microbiota is partially responsible for the metabolic disorders caused by residual tylosin.

### Exposure to TMDI dose of tylosin in early life is sufficient to cause lasting metabolic complications.

**FIG 3:**
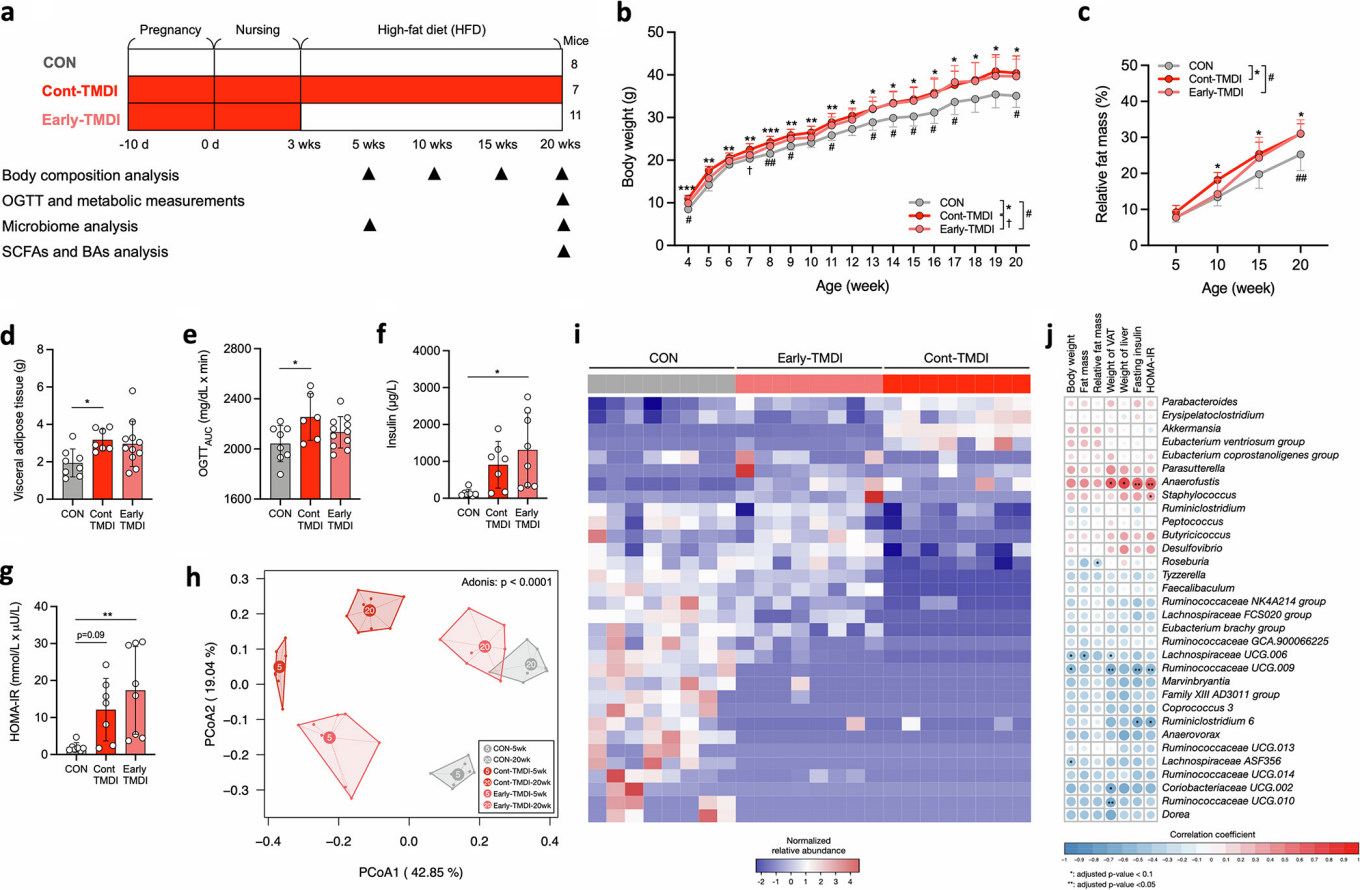
Early-life exposure to tylosin residue sufficiently induces obesity and metabolic disorder disease and modifies gut microbiota composition by enhancing the restructuring of obesity-related genera with deep-rooted changes. (a) Experimental design of early-life exposure model. (b) Body weight change (*n* = 8, 7, and 11 mice per group). (c) Relative fat mass change (*n* = 8, 7, and 11 mice per group). (d) Weight of visceral adipose tissue (*n* = 8, 7, and 11 mice per group). (e) AUC derived from the OGTT (*n* = 8, 6, and 11 mice per group). (f) Fasting Insulin (*n* = 7, 7, and 8 mice per group). (g) HOMA-IR index (*n* = 7, 7, and 8 mice per group). (h) PCoA based on Bray-Curtis distances of gut microbiota at 5 and 20 weeks of age (*n* = 8 mice per group). (i) Heat map showing 32 bacterial genus with significant differences (*q* < 0.05) among groups and (j) their Spearman’s correlation with obesity-related variables (*n* = 8 mice per group). Data are means and SD. For panels b and c, statistical analyses were performed with one-way ANOVA with Tukey’s range test as follows: CON versus early-TMDI (#, *P* < 0.05; ##, *P* < 0.01), CON versus Cont-TMDI (*, *P* < 0.05; **, *P* < 0.01; ***, *P* < 0.001), or Cont-TMDI versus early-TMDI (†, *P* < 0.05). For panels d to g, one-way ANOVA with Tukey’s range test was performed (*, *P* < 0.05; **, *P* < 0.01). For panel h, Adonis was performed to test the difference among groups. For panel i, a Kruskal-Wallis test with an FDR-adjusted *P* value was performed (*q* < 0.05). For panel j, Spearman’s correlation was performed with an FDR-adjusted *P* value (*, *q* < 0.1; **, *q* < 0.05). Abbreviations: AUC, area under the curve; CON, control; HFD, high-fat diet; HOMA-IR, homeostatic model assessment of insulin resistance; OGTT, oral glucose tolerance test; TMDI, theoretical maximum daily intake.

We next sought to investigate the influence of early life exposure to tylosin TMDI on obesity-related phenotypes and the gut microbiota (Fig. 3a). Cont-TMDI mice, which were continuously exposed to a TMDI dose of tylosin throughout the experimental period, displayed continuously elevated body weight, relative fat mass, visceral fat mass, and OGTT_AUC_, compared with HFD-CON mice (Fig. 3b to e). Interestingly, early-TMDI mice, exposed to tylosin TMDI during pregnancy and the nursing period, also exhibited consistently elevated body weight, relative fat mass, fasting insulin, and HOMA-IR index values after cessation of tylosin exposure (Fig. 3b to g). These findings suggest that exposure to a residual dose of antibiotics from food early in life can have a long-lasting effect on metabolism, leading to obesity.

### Early exposure to TMDI dose of tylosin alter the abundance of specific bacteria related to the metabolic homeostasis of the host.

The overall gut microbiota composition based on PCoA of Bray-Curtis distances showed that tylosin influenced the gut microbiota composition at weeks 5 and 20 (Adonis; *P* < 0.0001) (Fig. 3h). Compared with CON mice, Cont-TMDI mice showed a greater difference than early-TMDI mice, suggesting that continuous tylosin exposure has a more significant effect than exposure restricted to early life (Fig. 3h). Furthermore, the early-TMDI and CON mice exhibited greater differences at week 5 than week 20, possibly due to the establishment of the gut microbiota (Fig. 3h).

The microbial colonization can be perturbed by exposure to antibiotics early in life, which, in turn, contributes to metabolic disorders in adulthood (36, 37). Thus, we propose that although the overall gut microbiota composition can be restored in later life, the abundance of specific bacteria associated with metabolic homeostasis of the host is persistently depleted. To investigate this hypothesis, we identified 32 bacterial genera significantly altered by early or continuous exposure to TMDI dose of tylosin (Fig. 3i), then examined the association between these bacterial genera and obesity-related phenotypes (Fig. 3j). Bacterial genera increased in both early-TMDI and Cont-TMDI mice, including *Anaerofustis*, and demonstrated a significant positive correlation with obesity-related phenotypes (Fig. 3i). In contrast, genera depleted in early-TMDI and Cont-TMDI mice, including bacteria belonging to the families *Lachnospiraceae* and *Ruminococcacea*e, exhibited a significantly negative correlation with obesity-related phenotypes (Fig. 3i). Notably, despite the discontinuation of tylosin exposure in early-TMDI mice at 3 weeks of age, several bacterial genera that are negatively correlated with obesity-related phenotypes remained diminished at week 20. That is, although early exposure to a residual dose of antibiotics may not have a significant impact on the overall microbial composition in later life, early depletion of certain bacteria that remain lost for the life of the host may contribute to metabolic dysfunctions in adulthood.

### Exposure to tylosin TMDI dose alters the composition of SCFAs and the conversion of bile acids with downstream effects on the FGF15 signaling pathway.

**FIG 4:**
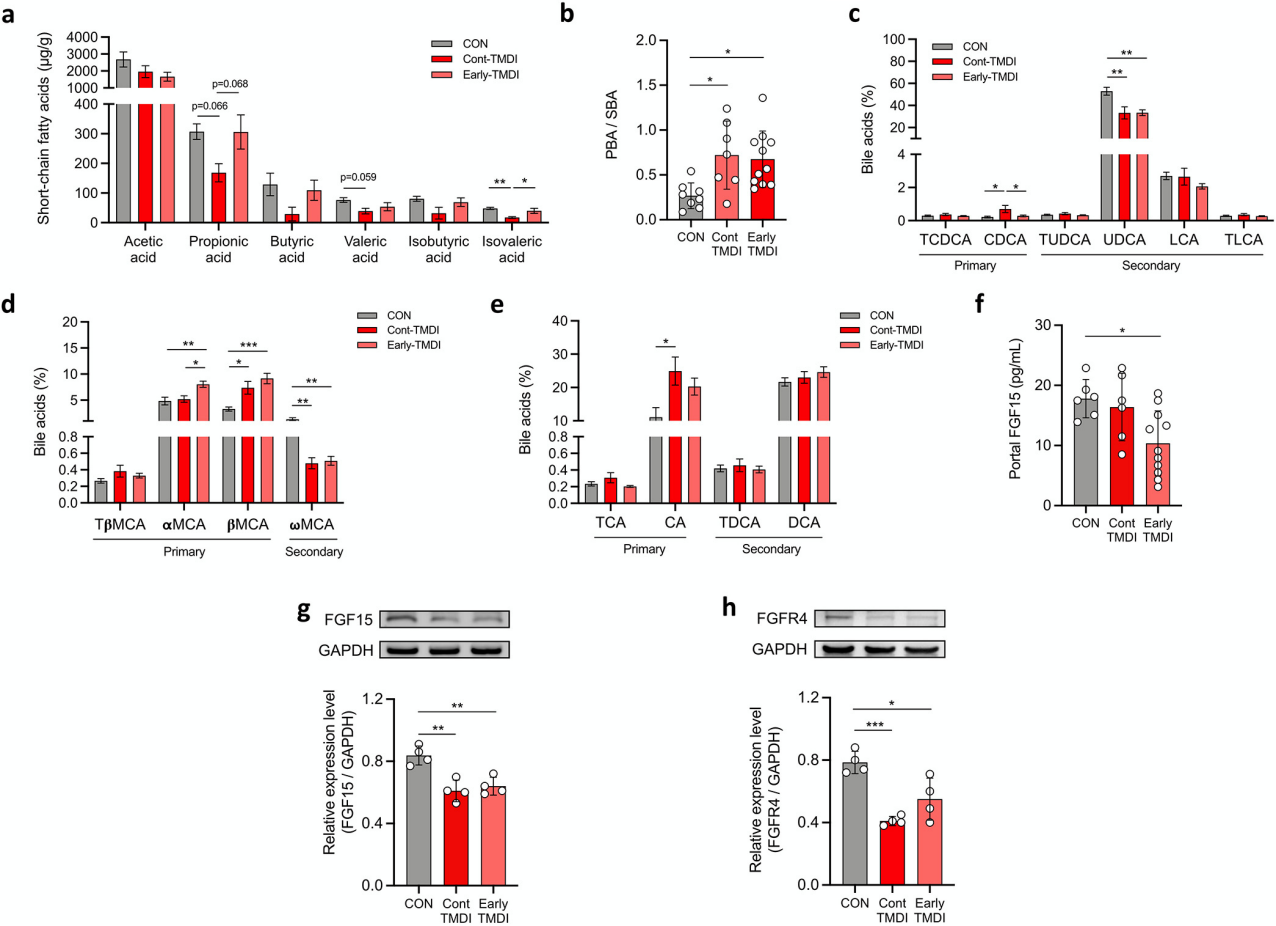
The modification of the fecal primary-secondary bile acid ratio by the gut microbiota and downregulation of the FGF15 signaling pathway are involved in the obesogenic and metabolic dysfunctions caused by tylosin residue exposure at TMDI dose. (a) Fecal short-chain-fatty-acid levels (*n* = 7 mice per group). (b) Ratio of fecal primary bile acids to secondary bile acids (*n* = 8, 7, and 11 mice per group). Levels of (c) non-12-OH bile acids, (d) muricholic acids, and (e) 12-OH bile acids (*n* = 8, 7, and 11 mice per group). (f) Portal-vein FGF15 levels (*n* = 6, 6, and 11 mice per group). (g) Western blotting of ileal FGF15 expression normalized to GAPDH (*n* = 4 mice per group). (h) Western blotting of hepatic FGFR4 level normalized to GAPDH (*n* = 4 mice per group). For a, c, d and e, data are means and SEM. For b, f, g and h, data are means and SD. For panels a to h, statistical analyses were performed with one-way ANOVA with Tukey’s range test (*, *P* < 0.05; **, *P* < 0.01; ***, *P* < 0.001). Abbreviations: AGP, antibiotic growth promoter; α-MCA, α-muricholic acid; β-MCA, β-muricholic acid; ω-MCA, ω-muricholic acid; CON, control; CDCA, chenodeoxycholic acid; FGF15, fibroblast growth factor 15; FGFR4, fibroblast growth factor receptor 4; GAPDH, glyceraldehyde 3-phosphate dehydrogenase; LCA, lithocholic acid; PBA, primary bile acid; SBA, secondary bile acid; T-β-MCA, tauro-beta-muricholic acid; TCDCA, taurochenodeoxycholic acid; TLCA, taurolithocholic acid; TMDI, theoretical maximum daily intake; TUDCA, tauroursodeoxycholic acid; UDCA, ursodeoxycholic acid.

Given that antibiotics reportedly alter SCFAs, bile acid composition, and their signaling pathways, thereby leading to metabolic consequences (15, 17), we next investigated the effect of the TMDI dose of tylosin on these two major metabolites. SCFA analysis showed that propionic acid and butyric acid, two main SCFAs associated with enhanced intestinal barrier function and insulin sensitivity (12, 38), exhibited decreasing trends in Cont-TMDI mice (Fig. 4a). Isovaleric acid, a branched SCFA reported to improve insulin-stimulated glucose uptake and enhance insulin sensitivity (39), was significantly decreased in Cont-TMDI mice (Fig. 4a). In addition, the abundance of nine ASVs assigned to the families *Lachnospiraceae* and *Ruminococcaceae*, which have been reported to be important for SCFA production, was significantly reduced in early-TMDI and Cont-TDMI mice compared to that in CON mice (Fig. 3i and j) (40, 41).

The total amount of bile acids and the ratio of conjugated to unconjugated bile acids were not significantly altered by tylosin exposure (Fig. S7a and b). Notably, the ratio of primary bile acids (PBA) to secondary bile acids (SBA) was significantly increased in Cont-TMDI and early-TMDI mice (*P* = 0.0177 and *P* = 0.0176, respectively) (Fig. 4b). Indeed, a decreased PBA/SBA ratio in NAFLD patients who have undergone bariatric surgery is associated with improved insulin sensitivity, indicating that an increased PBA/SBA ratio tends to induce metabolic disorder (42, 43). To further investigate the effect of tylosin on the conversion of PBA to SBA, the detected bile acids were classified into non-12-OH bile acids (Fig. 4c), muricholic acids (MCA) (Fig. 4d), and 12-OH bile acids (Fig. 4e) based on their metabolic pathways (44). The levels of PBAs, namely, chenodeoxycholic acid, α-MCA, β-MCA, and cholic acid (Fig. 4c to e), were significantly increased, whereas those of the SBA, including ursodeoxycholic acid (UDCA) and ω-MCA (Fig. 4c and d), were decreased in tylosin-treated mice, possibly due to inhibition of bacteria associated with epimerization and dehydroxylation of PBA, such as *Clostridia* and *Peptostreptococcus* (45) (Fig. S6c and d).

10.1128/msystems.00172-22.7 FIG S7 Effects of tylosin TMDI on bile acid composition and bacteria involved in the conversion of primary bile acids to secondary bile acids. (a) Total bile acid level in the cecum (*n* = 8, 7, and 11 mice per group). (b) Ratio of conjugated bile acids to unconjugated bile acids (*n* = 8, 7, and 11 mice per group). Relative abundance of bacteria exhibiting bile acid hydroxylase, 7α-dehydroxylase, and C-7 epimerase activity, including (c) Clostridia and (d) *Peptostreptococcus* (*n* = 8 mice per group). Data are means and SD (a and b) or means and SEM (c and d). Statistical analyses were performed by one-way ANOVA with Tukey’s range test (*, *P < *0.05; **, *P < *0.01; ***, *P < *0.001). Abbreviations: CBA, conjugated bile acid; CON, control; TMDI, theoretical maximum daily intake; UBA, unconjugated bile acid. Download FIG S7, TIF file, 0.2 MB. Copyright © 2022 Chen et al. 2022 Chen et al. https://creativecommons.org/licenses/by/4.0/ This content is distributed under the terms of the Creative Commons Attribution 4.0 International license.

Antibiotics have also been found to increase the PBA/SBA ratio with a subsequent decrease in plasma fibroblast growth factor 19 (FGF19; human orthologue of FGF15). FGF19 is an insulin-like hormone secreted by intestinal epithelium to regulate hepatic lipid and glucose metabolism by binding to FGFR4 in the liver, thereby decreasing peripheral insulin sensitivity (15, 46, 47). Moreover, *Parasutterella*, the genus enriched in tylosin-treated mice (Fig. 2e; Fig. S4 and S5), reportedly increases β-MCA and decreases ileal *Fgf15* gene expression (31). Hence, the FGF15/FGFR4 signaling pathway was further explored. Tylosin reduced ileal FGF15 expression in Early-TMDI and Cont-TMDI mice (*P* = 0.0016 and *P* = 0.0041, respectively) (Fig. 4g) and portal-vein FGF15 levels (*P* = 0.0214) (Fig. 4f) with subsequent reduction in hepatic FGFR4 levels (Fig. 4h). Collectively, tylosin TMDI-treated mice showed an increased PBA/SBA ratio, as well as lower FGF15 levels in the ileum and portal vein, and decreased expression of hepatic FGFR4, which may cause metabolic disorders by affecting metabolism-related signaling pathways in the liver (46–48).

## DISCUSSION

**FIG 5:**
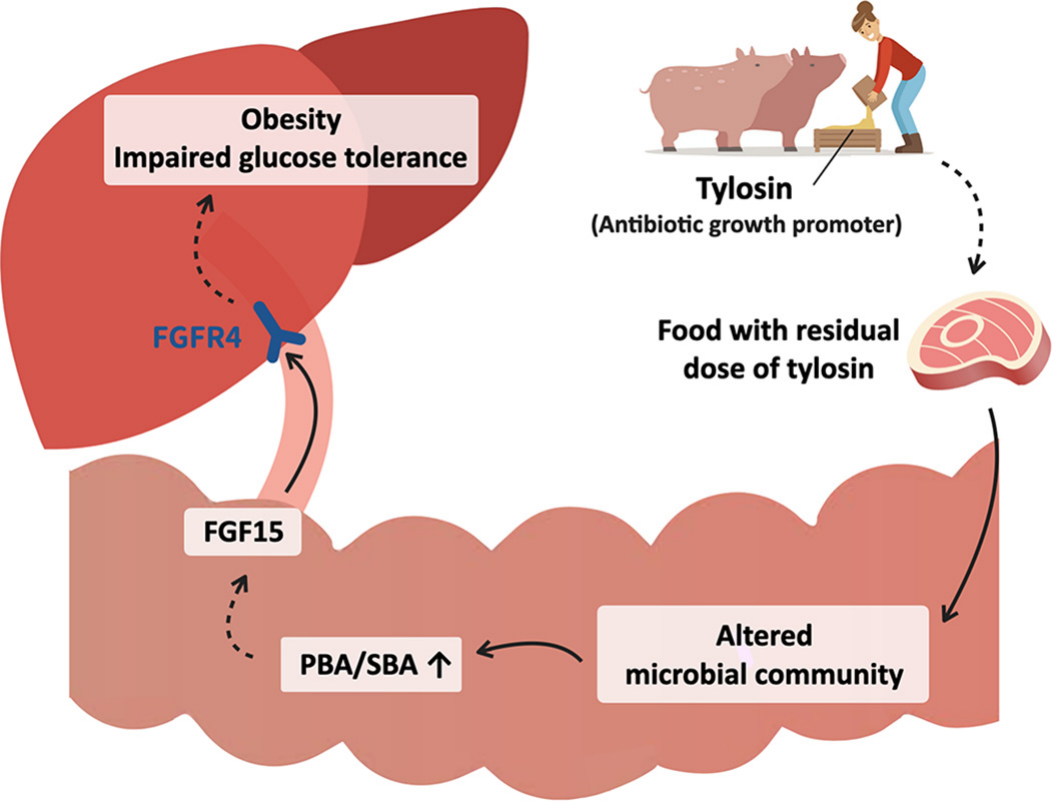
A residual dose of antibiotic growth promoter exacerbates HFD-induced metabolic disorder by altering the gut microbiota, microbial metabolites, and downstream signaling pathway. Abbreviations: AGP, antibiotic growth promoter; FGF15, fibroblast growth factor 15; FGFR4, fibroblast growth factor receptor 4; PBA, primary bile acid; SBA, secondary bile acid.

Continuous exposure to tylosin at ADI and TMDI doses perturbed the gut microbiota composition and facilitated HFD-induced metabolic complications. Moreover, the gut microbiota from tylosin-treated mice was able to cause similar obesogenic and metabolic phenotypes in germfree mice, suggesting that the gut microbiota play an important role in these metabolic complications. Since the gut microbial community is dynamic and susceptible to environmental shifts in early life, exposure to residual doses of tylosin at the early life stage was sufficient to persistently deplete specific bacteria involved in the metabolic homeostasis of the host, potentially inducing lasting adverse metabolic consequences. One of the mechanisms of action of the altered gut microbiota by residual tylosin is involved in the bile acid modification with downstream effects on the FGF15 signaling pathway (Fig. 5).

Several studies have examined the effects of different classes, different doses, and the timing of administration of antibiotics on host metabolic and obesogenic phenotypes. The phenotypic outcomes reported in these studies are inconsistent. In rodent models, a higher therapeutic dose of antibiotic tends to induce weight loss due to a considerable reduction in microbial population (49). In contrast, subtherapeutic doses of penicillin tend to result in weight gain, which predominately increases the fat mass (19, 24, 50). Interestingly, tylosin at a growth promotion dose increased the weight gain and lean content in swine (51), whereas a TMDI dose, the dose that humans could be exposed to, increased the fat mass and decreased the lean mass in mice in the present study. The inconsistency with respect to the effects of tylosin on host metabolism is possibly due to the different doses, classes, duration, and experimental animals used. Our study first demonstrates that continuous exposure to a residual dose of tylosin, which was 20-fold lower than that of low-dose penicillin (LDP) and up to 3-fold lower than the growth-promoting dose of tylosin used in food animals (19, 52), is sufficient to induce the development of obesogenic and metabolic phenotypes.

Continuous perturbation of the gut microbiota by antibiotics could contribute to obesity (19, 50). Our study demonstrates that the tylosin TMDI dose-altered microbial population can induce an obesogenic phenotype in germfree recipient mice (Fig. 2g to k). In addition, the abundance of specific bacterial taxa altered by tylosin correlates with obesity-related phenotypes (Fig. 3i and j). For instance, *Anaerofustis*, a tylosin-enriched bacterium, was found to be increased in obese humans (53). In contrast, the tylosin-depleted *Ruminococcaceae* and *Lachnospiraceae* were associated with a lower longitudinal weight gain (54). These results suggest that tylosin could cause an imbalance of the gut microbiome. Moreover, certain shifts of specific microbes could be associated with obesity.

The gut microbiota development in the neonatal period is critical to the host’s metabolism throughout a lifetime (19, 37). Early-life antibiotic exposure can lead to lasting effects on the host metabolism (28, 55, 56). A previous study found that exposure to LDP only 4 weeks after birth results in obesity in adulthood even if the antibiotic treatment is interrupted (19, 57). In this study, despite early-TMDI mice exhibiting a microbial community more closely resembling that of CON mice at week 20 than at week 5 (Fig. 3h), their fat mass at week 20 was more significantly increased than at week 5 (Fig. 3c). Early colonization by commensal bacteria is necessary for the maturation of host immunity; thus, antibiotics could disturb gut microbiome colonization and immune system development, associated with long-term metabolic dysfunction (37).

The other possible mechanism of tylosin TMDI dose on metabolic phenotype is the shaping of microbial metabolites and their signaling pathways. Our metabolomic analysis revealed that the TMDI dose of tylosin modified both SCFAs and bile acids. Cont-TMDI mice showed decreased isobutyric acid and isovaleric acid, which reportedly improve insulin-stimulated glucose uptake and enhance insulin sensitivity (39). Additionally, Cont-TMDI mice showed a reducing trend of butyric acid and propionic acid, which were associated with enhanced intestinal barrier function and insulin sensitivity (12, 38). Reduction of SCFAs could have contributed to the depletion of *Lachnospiraceae* and *Ruminococcaceae* in early-TMDI and Cont-TMDI mice (53), and the observed increased PBA/SBA ratio may be caused by the broad inhibition of specific bacteria related to the conversion of PBA (Fig. 5).

Increased PBA/SBA ratio has been associated with decreased insulin sensitivity in patients with NAFLD or nonalcoholic steatohepatitis (42, 58). In this study, tylosin TMDI-treated mice showed increased PBA/SBA ratios and lower FGF15 levels in the ileum and portal vein, thereby decreasing the expression of hepatic FGFR4, which may cause metabolic disorders by affecting metabolism-related signaling pathways in the liver (46–48). Consistent with these observations, antibiotics were reported to increase the PBA/SBA ratio, with subsequently decreased plasma FGF19 (human orthologue of FGF15) and peripheral insulin sensitivity (15).

In conclusion, exposure to antibiotic residues in food may disturb the gut microbiota community and alter related metabolites, exacerbating obesity and metabolic dysfunction. This study provides valuable insights into the residual antibiotics, gut microbiota, and host metabolic phenotype interaction. Our finding could support the future direction of food safety issues on the regulation of permissible exposure levels of antibiotic residues. Of note, the safe thresholds (ADI/TMDI) of exposure should be re-established and implemented by considering the impact of antibiotics on the gut microbiota.

## MATERIALS AND METHODS

### Antibiotic selection and dose calculation.

Tylosin was selected as a model AGP due to its high annual consumption as a veterinary antibiotic (Bureau of Animal and Plant Health Inspection and Quarantine, 2014) and its presence in animal-derived food (59–61). The ADI and TMDI doses were obtained from the World Health Organization Technical Report Series (62).

### Experimental design of the animal studies.

Animal experiments were performed with permission from the Institutional Animal Care and Use Committee of National Taiwan University (approval numbers NTU-106-EL-051 and NALC 107-0-006-R2). All mice were purchased from the National Laboratory Animal Center (Taipei City, Taiwan). For animal studies I and III, the number of offspring mice in each experimental group varied according to the number of offspring borne by the mother mice (3 mother mice per group).

### (i) Antibiotic residue exposure study.

C57BL/6J mother mice (*n* = 3 per group) received tylosin at ADI (0.37 mg/kg) or TMDI (0.047 mg/kg) doses in drinking water during pregnancy approximately 10 days before giving birth. Only male offspring mice were used in this study. To investigate the effect of tylosin in different types of diet (NCD and HFD), the male offspring mice were randomly divided into groups receiving NCD (MFG; Oriental Yeast Co., Ltd., Tokyo, Japan) and HFD (60% kcal from fat) (D12492; Research Diets, New Brunswick, NJ, USA) and continuously supplied with the tylosin after weaning until 20 weeks old. Control mice (CON) did not receive antibiotics. The sizes of the NCD-CON, NCD-ADI, NCD-TMDI, HFD-CON, HFD-ADI, and HFD-TMDI groups were 12, 12, 8, 12, 12, and 12, respectively. Body composition was measured at 8, 12, and 17 weeks. The metabolic measurements and OGTT were performed at 20 weeks prior to sacrifice. After euthanasia by carbon dioxide inhalation, blood and tissue samples were collected and stored at −80°C.

### (ii) Fecal microbial transplantation study.

Eight-week-old C57BL/6J germfree mice were randomly divided into fecal microbiota transplantation control (FMT-CON; *n* = 10) and FMT-TMDI (*n* = 10) groups, which were transplanted with fecal microbiotas from HFD-CON and HFD-TMDI mice, respectively. The fecal microbiotas of HFD-CON and HFD-TMDI mice in animal experiment I were prepared by pooling the feces content and mixing with phosphate-buffered saline (PBS) before being administered to the germfree mice. The fecal microbiota was supplied by gavage twice to the germfree mice on day 0 and day 3. After FMT, the recipient mice were housed in two independent isolators and fed with irradiated HFD until 20 weeks of age. Before the recipient mice were euthanized, the body composition analysis and OGTT were performed.

### (iii) Early-life exposure study.

The experimental design of the early-life exposure study was similar to the experimental animal design except for the duration of tylosin exposure. In the tylosin-treated groups, C57BL/6J mother mice received tylosin at the TMDI dose (0.047 mg/kg) in drinking water during pregnancy approximately 10 days before giving birth. Only male offspring were used for the study. In the early-TMDI group (*n* = 11), the duration of tylosin exposure was limited to the gestation and lactation periods; in the Cont-TMDI group (*n* = 7), tylosin was continuously supplied from pregnancy to 20 weeks of age; in the CON group (*n* = 8), there was no exposure to tylosin residue. Body composition was measured at 5, 10, 15, and 20 weeks. The OGTT was performed before the mice were euthanized at 20 weeks.

### Body composition analysis.

Body composition was determined using a Minispec LF50 TD-NMR body composition analyzer (Bruker, Billerica, MA, USA), which provides the measurement of body weight and lean and fat mass. The relative fat mass was calculated as the fat mass (g)/body weight (g) ratio.

### Glucose and insulin sensitivity.

For the OGTT, mice were deprived of food for 5 h. Blood was collected from the submandibular vein, and the glucose levels were measured using a glucometer (Roche, Basel, Switzerland) at 0, 15, 30, 60, 90, and 120 min after oral administration with 2 g/kg glucose. The fasting insulin levels were detected by an enzyme-linked immunosorbent assay (ELISA) kit (Mercodia, Uppsala, Sweden). The HOMA-IR index was calculated using the following formula: fasting glucose (nmol/L) × fasting insulin (μU/mL)/22.5 (63).

### Histopathological analysis of liver and adipose tissue.

Liver and adipose tissue sections were collected and fixed in 10% formalin solution. Histopathological analysis was performed by the formalin-fixed, paraffin-embedded, and hematoxylin and eosin (H&E)-stained slide method. The fatty liver score was estimated by a pathologist as previously described (64). The fatty liver score included the evaluation of steatosis (macrovesicular, microvesicular, and hypertrophy) and inflammation (number of inflammatory foci). The visceral adipocyte quantification was performed by HCImage Live software (HCImage, Sewickley, PA, USA).

### Quantification of plasma lipopolysaccharides.

Plasma samples were centrifuged and added to HEK-Blue mTLR4 cells in HEK-Blue detection medium (InvivoGen, San Diego, CA, USA). After 24 h of incubation, the color of secreted embryonic alkaline phosphatase (SEAP) released by the reporter cells was measured by a spectrophotometer at 620 nm (65).

### Metabolic measurements.

Metabolic measurements including food intake, locomotor activity, oxygen consumption (VO_2_), and carbon dioxide production (VCO_2_) were made using the Promethion metabolic phenotyping system (Sable Systems, Las Vegas, NV, USA). Monitoring was performed for 24 to 48 h with *ad libitum* access to food and water after mice had been acclimatized to cages for 6 to 12 h.

### Fecal microbiota extraction and 16S rRNA gene sequencing.

The mice in each group were randomly selected for fecal microbiota analysis. Mouse feces were collected and frozen immediately at −80°C. Fecal DNA extraction and library preparation and sequencing were performed according to the protocol provided by Qiagen (Germantown, MD, USA). Briefly, fecal microbial DNA was isolated by using a QIAamp 96 PowerFecal QIAcube HT kit (Qiagen, Germantown, MD, USA). The 16S V4 region was amplified by the forward primer F515 (5′-GTGCCAGCMGCCGCGGTAA-3′) and the reverse primer R806 (5′-GGACTACHVGGGTWTCTAAT-3′). The reaction conditions were as follows: 3 min at 95°C, followed by 25 cycles of 95°C for 30 s, 55°C for 30 s, and 72°C for 30 s, and 5 min at 72°C for a final extension. For library construction, the amplified PCR product was attached with Illumina sequencing adapters with a Nextera XT Index kit and then purified using AMPure XP beads. The library quantification was conducted using the DNA 1000 kit and 2100 Bioanalyzer instrument (Agilent Technologies, Santa Clara, CA, USA). The sequencing (paired-end reads, 2 × 150 bp) was performed by Illumina NextSeq and Illumina MiSeq (Illumina, San Diego, CA, USA).

### Bioinformatic analysis for microbial taxonomic profiling.

The amplicon sequences were processed by a QIIME 2 pipeline (version 2019.10, https://qiime2.org). The primer sequences of raw reads were trimmed using the cutadapt plugin. The trimmed single-end (forward) sequences were subsequently denoised with the DADA2 plugin of QIIME2 (66). To obtain qualified data, we truncated reads to 130 bp from the 3′ end based on the quality score. The DADA2 outputs of high-confidence ASVs went through quality filtering, denoising, and chimeric-read removing. The classify-consensus-vsearch plugin was applied for taxonomy assignment by aligning against SILVA 132 99% 16S rRNA gene sequences, and the identity cutoff was set at ≥80% sequence similarity by default. The vegan package in R was used to calculate Shannon index and perform principal-coordinate analysis (PCoA) based on the Bray-Curtis distance (67). The permutation multivariate analysis of variance (ANOVA) using distance matrixes (Adonis) was performed to calculate the significance of microbiota composition among groups. The PCoA plots for gut microbiotas with an association with obesity-related features of 17-week-old HFD-fed mice were performed by the envfit function in the vegan R package, displaying as the vectors (*P* < 0.05). ALDEx2 was performed by R (29).

### Correlation analysis.

The Spearman’s correlation analysis between PCoA1 and PCoA2 of gut microbiota against individual obesity-related parameters was performed with the false discovery rate (FDR)-adjusted *P* value. The correlation coefficient and *P* value of Spearman’s correlation between PC1_obesity biomarkers_ (representing the overall obesity phenotype) and PCoA2_microbiota_ (representing the gut microbiota composition) was calculated.

### PCA of obesity biomarkers.

Principal-component analysis (PCA) was used for dimension reduction of obesity biomarkers (27), including body weight at 17 weeks of age, fat mass at 17 weeks of age, relative fat mass at 17 weeks of age, weight gain between 3 to 17 weeks of age, fat mass gain between 8 to 17 weeks of age, fasting glucose, OGTT_AUC_, fasting insulin, HOMA-IR, weight of epididymal adipose tissue (eWAT), and fatty liver score.

### Fecal short-chain fatty acid analysis.

The SCFAs we analyzed included acetic acid (C_2_), propionic acid (C_3_), butyric acid (C_4_), isobutyric acid (C_4_), valeric acid (C_5_), and isovaleric acid (C_5_). Briefly, 20 mg of the raw feces was dissolved in 500 μL of a 0.5% H_3_PO_4_ aqueous solution and homogenized with a Geno/Grinder instrument at 1,000 rpm for 2 min. The sample solutions were then centrifuged at 18,000 × *g* for 10 min at 4°C to separate deposits. Afterwards, 285 μL of the supernatant was collected in a clean centrifuge tube, and 15 μL of acetate-d3 was added. For the liquid-liquid extraction of SCFAs, 300 μL of butanol and the final solution were mixed and centrifuged. Last, 20 μL of internal standard propionate-d5 was added to 180 μL of the upper organic layer. Gas chromatography-mass spectrometry (GC-MS) analysis was performed using an Agilent 7890A gas chromatograph (Agilent Technologies) coupled with a Pegasus 4D two-dimensional GC (GC×GC)–time-of-flight (TOF)–MS system (Leco Corporation, St. Joseph, MI, USA) using a VF-WAXms capillary column (68).

### Fecal bile acid analysis.

The bile acid quantification included four primary bile acids (α-MCA, β-MCA, CA, and UDCA), four secondary bile acids (ω-MCA, chenodeoxycholic acid [CDCA], deoxycholic acid [DCA], and lithocholic acid [LCA]), and seven conjugated bile acids (tauro-β-muricholic acid [T-β-MCA], tauroursodeoxycholic acid [TUDCA], taurocholic acid [TCA], glycocholic acid [GCA], taurochenodeoxycholic acid [TCDCA], taurodeoxycholic acid [TDCA], and taurolithocholic acid [TLCA]). Briefly, 20 mg of raw feces was dissolved in 200 μL of 70% d3-cholic acid aqueous solution containing 2 ppm d4-cholic acid as an internal standard, and it was homogenized using an ultrasonicator for 30 min. The sample solutions were then centrifuged at 18,000 × *g* for 5 min. LC-MS analysis was performed using an UltiMate 3000 liquid chromatography system (Thermo Fisher Scientific, Dreieich, Germany) coupled with a high-resolution Q Exactive Plus instrument equipped with an ESI source (Thermo Fisher Scientific) and an Acquity HSS T3 (2.1 by 100 mm, 1.7 μm) column (Waters, Milford, MA, USA).

### Biochemical analysis of the FGF15-FGFR4 pathway.

For Western blotting, frozen tissues were lysed in lysis buffer containing 7 M urea, 2 M thiourea, 2% CHAPS {3-[(3-cholamidopropyl)-dimethylammonio]-1-propanesulfonate}, 0.002% bromophenol blue, 60 mM dithiothreitol (DTT), and a protease and phosphatase inhibitor cocktail. The cell lysates were sonicated for 5 min and centrifuged at 17,500 × *g* for 30 min at 4°C. Total protein content was measured based on the Bradford assay with a Bradford reagent (Bioshop Canada Inc., Burlington, Canada) and an ELISA reader. For the loading buffer, 62.5 mM Tris-HCl, 10% glycerol, 2% SDS, and 0.01% bromophenol blue were mixed with the protein samples and then heated at 95°C for 10 min for protein denaturation. Proteins were separated by 10% or 12% SDS-polyacrylamide gel electrophoresis; they were then transferred onto polyvinylidene difluoride membranes (Millipore, Burlington, MA, USA). After blocking with 5% bovine serum albumin in Tris-buffered saline with Tween 20, the membranes were incubated with rabbit monoclonal anti-glyceraldehyde-3-phosphate dehydrogenase (GAPDH) antibody (1:5,000; catalog no. 5174; Cell Signaling, Danvers, MA, USA), or rabbit polyclonal anti-FGF15 antibody (1:500; catalog no. ab229630; Abcam, Cambridge, UK), or rabbit polyclonal anti-FGFR4 antibody (1:500; catalog no. ab119378; Abcam) at 4°C for 16 h and subsequently incubated with horseradish peroxidase (HRP)-linked anti-rabbit IgG antibody (1:3,000; catalog no. 7074; Cell Signaling). The protein expression signal was captured and quantified using the BioSpectrum AC imaging system (UVP, Upland, CA, USA) and ImageJ (version 1.53; National Institutes of Health, Bethesda, MD, USA), respectively (69). Portal-vein FGF15 levels were quantified using an ELISA kit (Mercodia, Uppsala, Sweden).

### Statistical analysis.

Data are presented as means and standard deviations (SD) or means and standard errors of the means (SEM). One-way ANOVA and Tukey’s range test or unpaired two-tailed Student’s *t* test were applied for intergroup comparisons. Statistical assessment of the gut microbiome, SCFAs, and bile acids was performed using the Kruskal-Wallis test with/without false discovery rate or one-way ANOVA with Tukey’s range test. All statistical data were analyzed using GraphPad Prism software (version 9.2.0; GraphPad Software, San Diego, CA, USA) or RStudio (version 1.2.5001, RStudio, Boston, MA, USA).

### Data availability.

The raw 16S rRNA sequencing data are accessible at the National Center for Biotechnology Information Short Read Archive (BioProject no. PRJNA715326).
