# Supplementary figures and images for: Dietary Exposure to Antibiotic Residues Facilitates Metabolic Disorder by Altering the Gut Microbiota and Bile Acid Composition
Source: mSystems. 2022 Jun 7;7(3):e00172-22. doi: 10.1128/msystems.00172-22 (PMC9239188; doi:10.1128/msystems.00172-22)

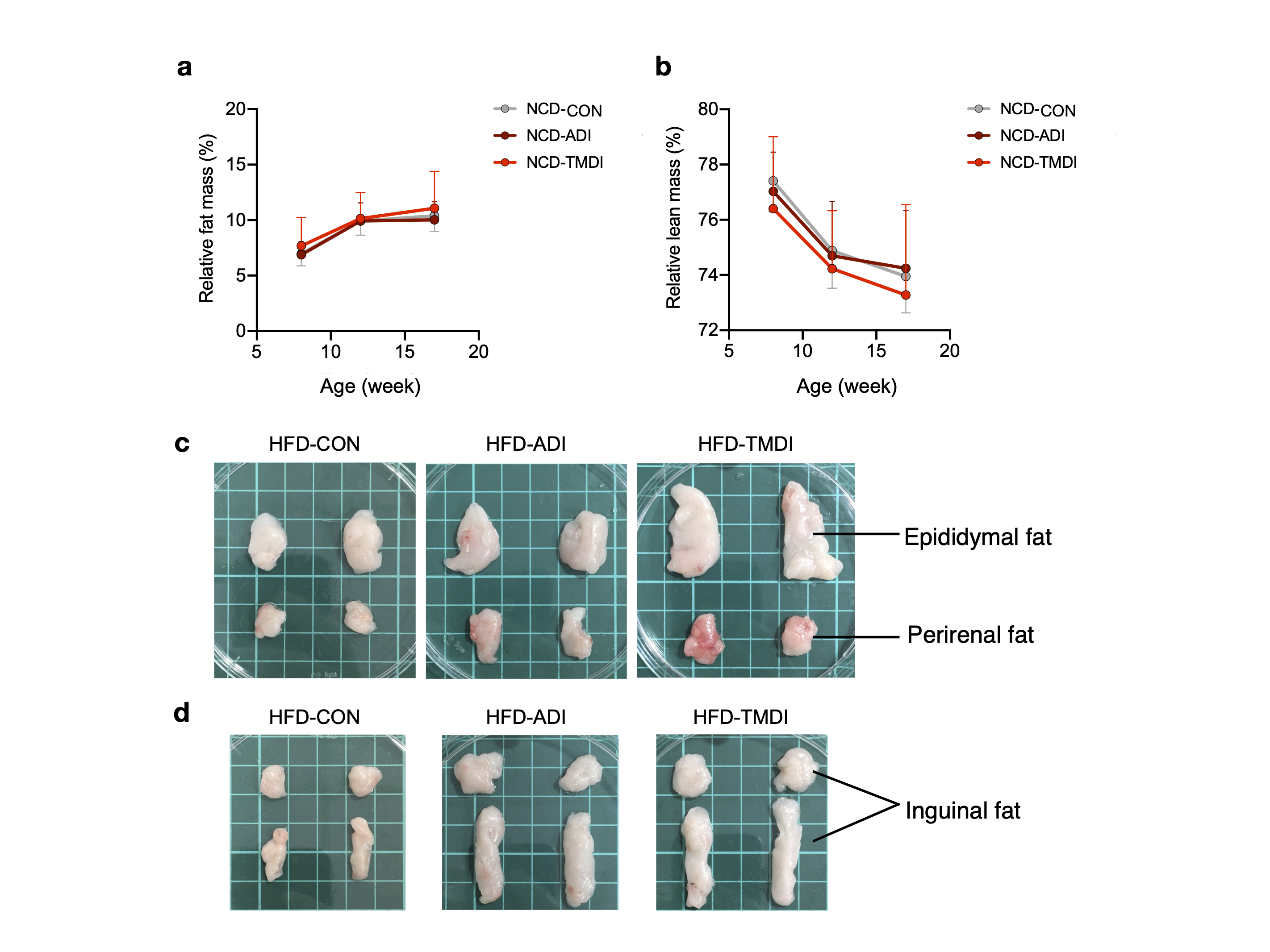

Supplement: FIG S1 [file msystems.00172-22-sf001.tif]

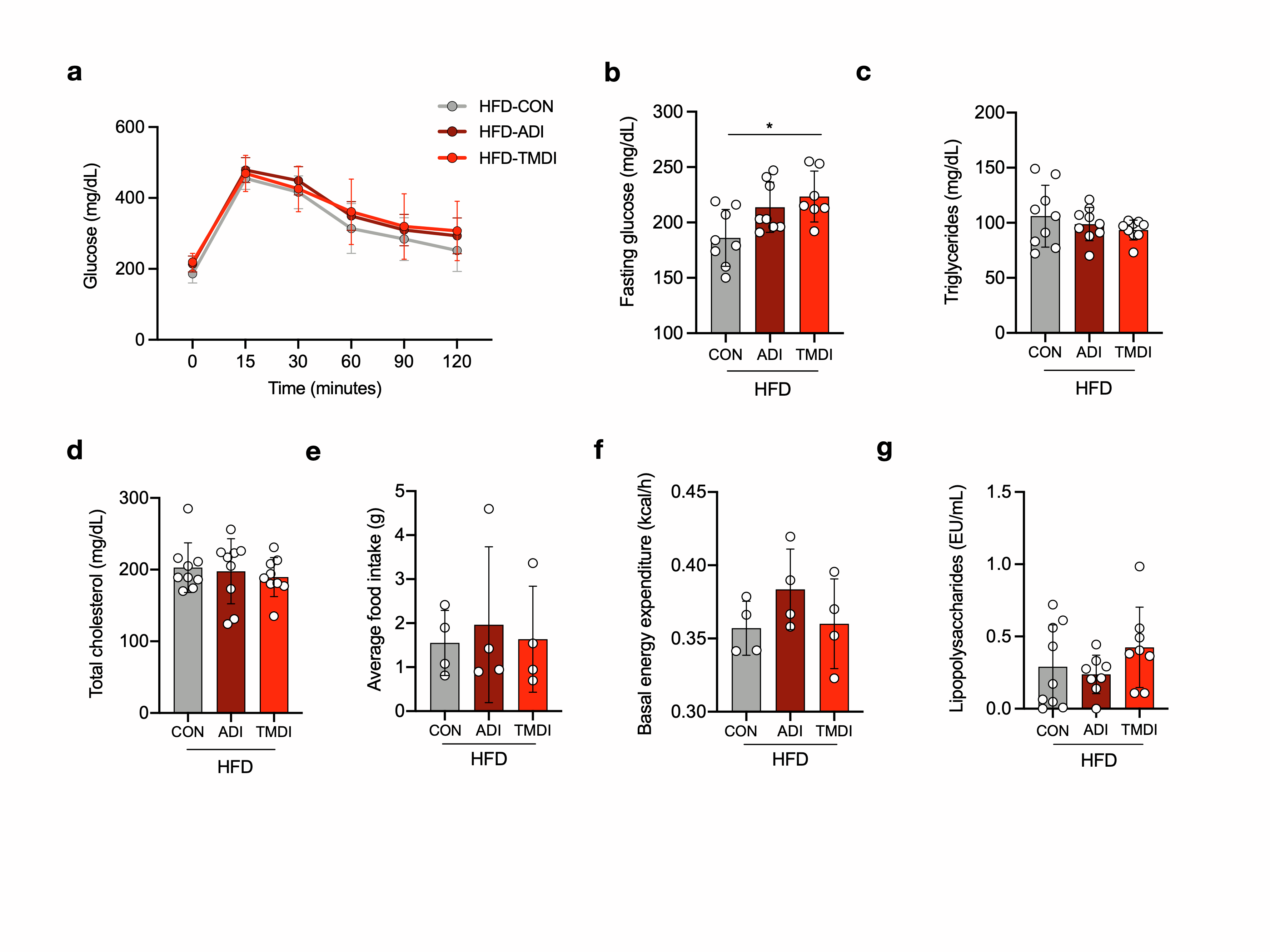

Supplement: FIG S2 [file msystems.00172-22-sf002.tif]

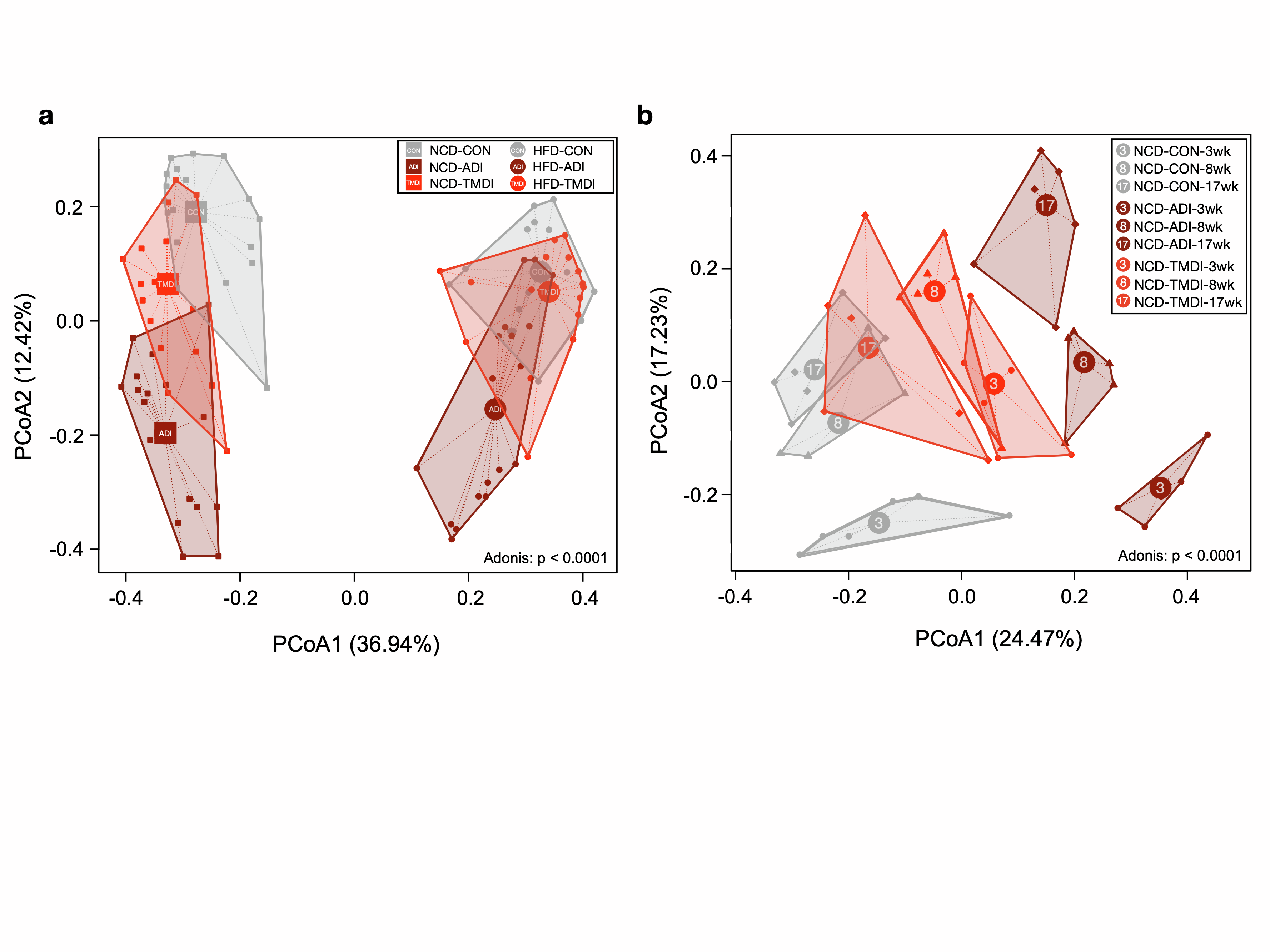

Supplement: FIG S3 [file msystems.00172-22-sf003.tif]

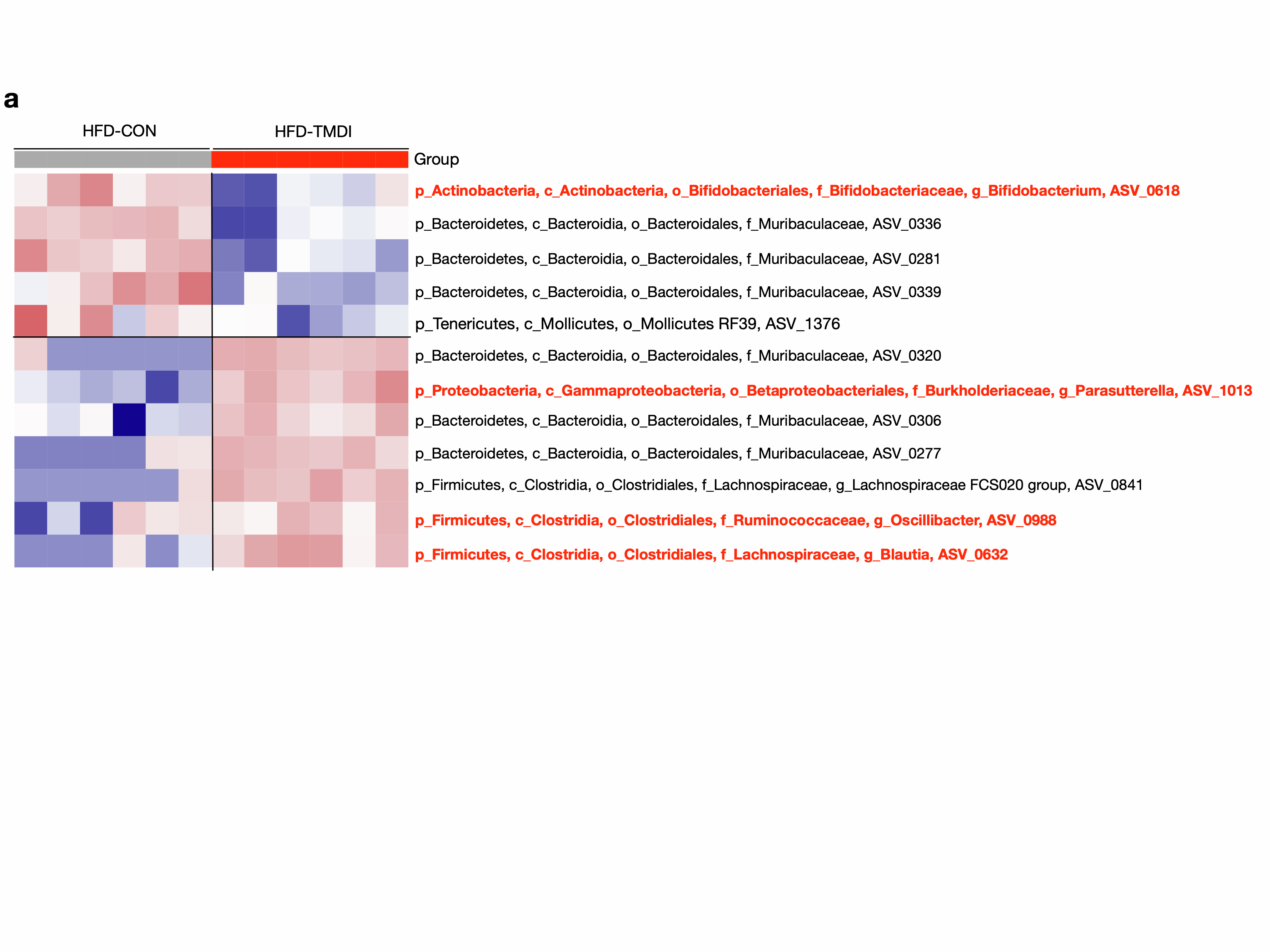

Supplement: FIG S4 [file msystems.00172-22-sf004.tif]

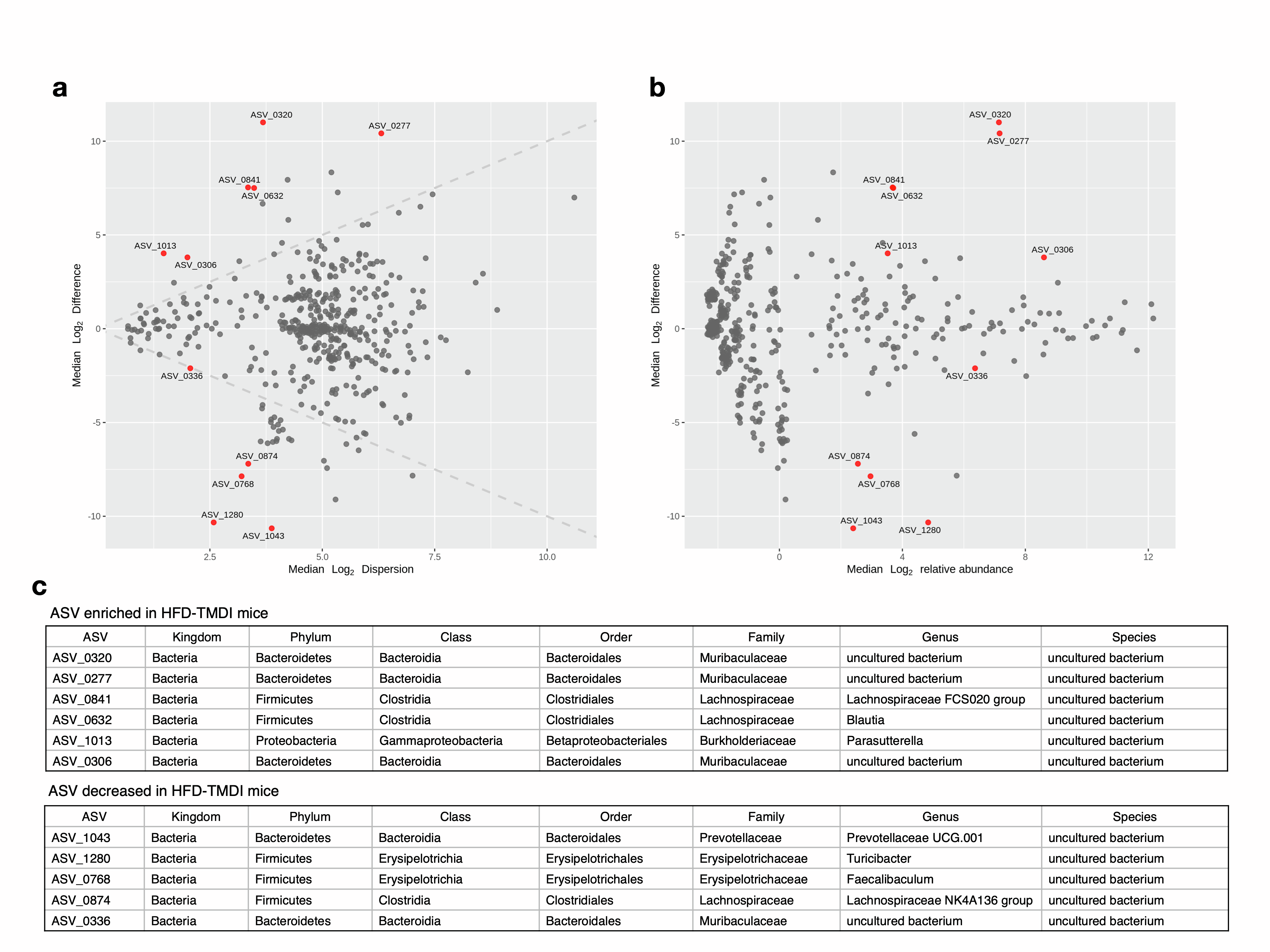

Supplement: FIG S5 [file msystems.00172-22-sf005.tif]

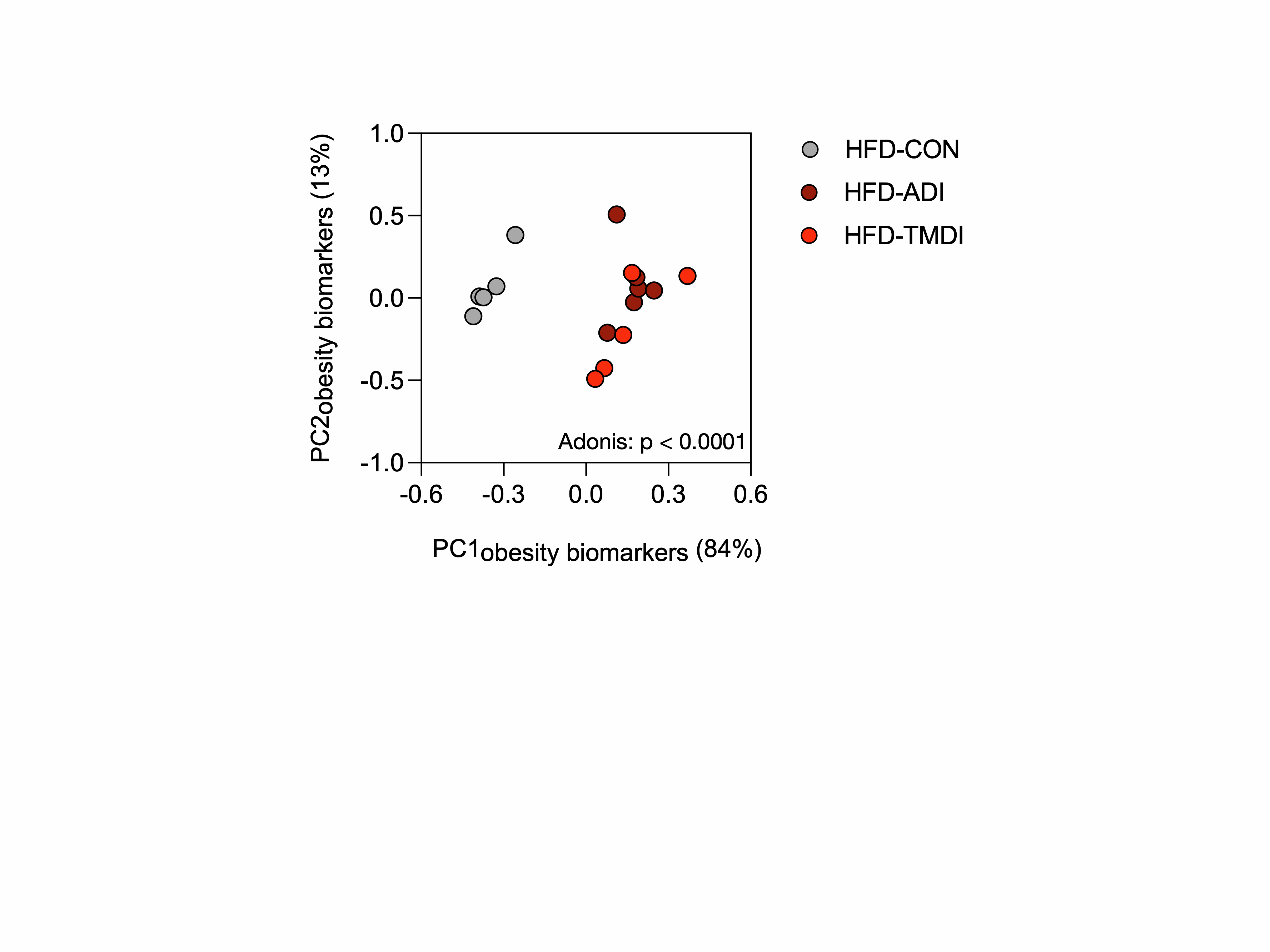

Supplement: FIG S6 [file msystems.00172-22-sf006.tif]

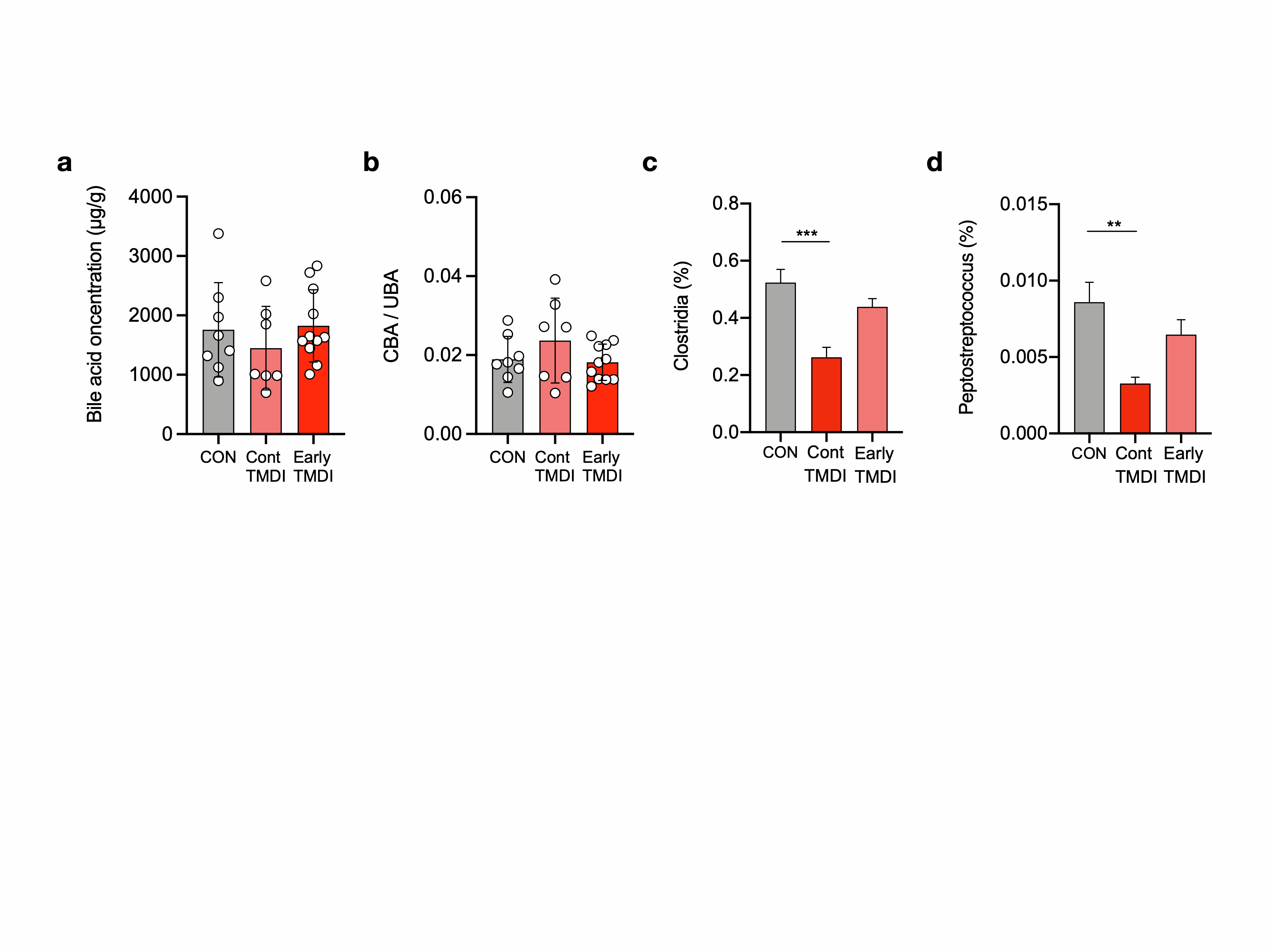

Supplement: FIG S7 [file msystems.00172-22-sf007.tif]
