# Supplementary material for: Dietary Exposure to Antibiotic Residues Facilitates Metabolic Disorder by Altering the Gut Microbiota and Bile Acid Composition
Source: mSystems. 2022 Jun 7;7(3):e00172-22. doi: 10.1128/msystems.00172-22 (PMC9239188; doi:10.1128/msystems.00172-22)
Supplement: TABLE S1 [file msystems.00172-22-st001.docx]

| Obesity-related parameters^b^ |  | PCoA1^a^ | | |  | PCoA2^a^ | | |
| --- | --- | --- | --- | --- | --- | --- | --- | --- |
|  |  | *r* | *p* | *q* |  | *r* | *p* | *q* |
| Body weight |  | -0.374 | 0.155 | 0.243 |  | 0.441 | 0.089 | 0.098 |
| Fat mass |  | -0.453 | 0.080 | 0.185 |  | 0.694 | **0.004** | **0.014** |
| Relative fat mass |  | -0.497 | 0.052 | 0.185 |  | 0.726 | **0.002** | **0.011** |
| Fasting glucose |  | -0.538 | **0.034** | 0.185 |  | 0.479 | 0.062 | 0.082 |
| OGTTAUC |  | -0.329 | 0.213 | 0.292 |  | 0.424 | 0.104 | 0.104 |
| Fasting insulin |  | -0.126 | 0.641 | 0.705 |  | 0.588 | **0.019** | **0.038** |
| HOMA-IR |  | -0.200 | 0.456 | 0.558 |  | 0.665 | **0.006** | **0.017** |
| Fatty liver score |  | -0.534 | **0.035** | 0.185 |  | 0.579 | **0.021** | **0.038** |
